# Aluminum Plates

**Published:** 1904-02

**Authors:** John G. Harper

**Affiliations:** St. Louis.


### ALUMINUM PLATES.

By John G. Harper, D. D. S., St. Louis.

Aluminum plates give as good satisfaction as gold plates and are less in weight and expense. I know of plates that have been in use for sixteen years, and the fit and condition of the mouth is fine. Rubber plates affect the roof of the mouth and do not fit well after from five to seven years. In the long run aluminum plates are the cheapest and best that can be used.

The swaging of aluminum is easily done, requiring but one die. The model should be built up slightly with very thin sheet wax to relieve the hard ridge in the roof of the mouth, and trimmed near the posterior portion at the sides to insure close adaptation to the soft parts and exclusion of the air, thus giving good adhesion. After swaging, the plate is trimmed so as to allow a good depth to a rim of rubber extending above the edge of the plate. The rim is given a slight incline toward the alveolar ridge, this gives the rubber a firm hold on the plate. The most elevated portion of the plate over the ridge is barbed with a sharp-pointed instrument, making the barbs point in opposite directions. The plate should be tried in the mouth and made to fit properly. The model may be used, or an impression may be taken with the plate in position; this is necessary to build up the rim with wax, to be replaced by rubber above the edge of the metal plate. A roll of wax is now placed on the plate to secure the bite, and the wax is properly shaped to make the desired fullness under the lips and cheek. Proceed now the same as with a rubber plate. It is always better to try the plate in the mouth with the teeth arranged. After finishing the plate wipe exposed metal with caustic soda solution, which gives the aluminum a pleasing white surface.—*Dental Era.*

